# Reducing sickness absence among public-sector healthcare employees: the difference-making roles of managerial and employee participation

**DOI:** 10.1007/s00420-024-02048-0

**Published:** 2024-02-27

**Authors:** M. Akerstrom, J. Severin, E. J. Miech, E. Wikström, M. Roczniewska

**Affiliations:** 1 Region Västra Götaland, Institute of Stress Medicine, Carl Skottbergs Gata 22B, 413 19 Gothenburg, Sweden; 2 School of Public Health and Community Medicine, Institute of Medicine, The Sahlgrenska Academy at University of Gothenburg, Gothenburg, Sweden; 3 Center for Health Services Research, Regenstrief Institute, Indianapolis, IN USA; 4 Department of Business Administration, School of Business, Economics and Law, University of Gothenburg, Gothenburg, Sweden; 5 Procome Research Group, Department of Learning, Informatics, Management and Ethics, Medical Management Centre, Karolinska Institutet, Stockholm, Sweden; 6 Institute of Psychology, SWPS University, Sopot, Poland

**Keywords:** Process evaluation, Absenteeism, Workplace interventions, Organization, Work environment, Public sector, Organizational-level intervention, Coincidence analysis, Configurational comparative methods

## Abstract

**Purpose:**

Evaluations of organizational-level interventions to prevent work-related illness have identified enabling factors, but knowledge of necessary and sufficient conditions for intervention success is needed. The aim was to identify difference-making factors that distinguish intervention groups with and without a positive intervention effect on sickness absence.

**Methods:**

An organizational-level intervention designed to decrease sickness absence by providing support from process facilitators was implemented at eight healthcare workplaces in Sweden between 2017 and 2018. We applied coincidence analysis (CNA) to analyze 34 factors and determine which factors were necessary and sufficient for a successful implementation of tailored interventional measures on an organizational level (dichotomous) and reduced sickness absence (trichotomous).

**Results:**

Two factors perfectly explained both the presence and absence of a successful implementation: “a high sense of urgency” and “good anchoring and participation from the strategic management”. The presence of either of these factors alone was sufficient for successful implementation, whereas the joint absence of both conditions was necessary and sufficient for the absence of successful implementation and an intervention effect. In addition, high employee participation was both necessary and sufficient for a high intervention effect. For organizations without high employee participation, successful implementation led to a medium-effect size.

**Conclusions:**

This study identified participation as a difference-maker in the implementation process. Participation from different stakeholders turned out to be important in different phases. When implementing organizational-level interventions, high participation from both strategic management and employees appears to be crucial in terms of the intervention’s effect on sickness absence.

## Introduction

Access to a specially trained workforce is crucial for any healthcare organizations’ overall performance and factors on individual, workplace, and organizational levels have been shown to affect the employee turnover and sickness absence among health care employees (Daouk-Öyry et al. 2014). In Sweden, all 21 healthcare regions report a current shortage of healthcare professionals, especially midwives, specialist nurses, and physicians within general medicine and psychiatry. Furthermore, the absence of a healthy workforce has been shown to have negative effects on both the quality of care and the financial outcomes within the healthcare sector (Gaudine and Gregory 2010; Homburg et al. 2009; Laschinger et al. 2009; Liu et al. 2012; McGillis Hall and Doran 2007).

To improve the work environment, interventions aiming to change the way the work is organized, designed, or managed (i.e., organizational-level interventions) have been suggested (Cox et al. 2007; Giga et al. 2003; Kompier 2001; Nielsen and Randall 2013; Nielsen et al. 2010). These interventions consist typically of multiple interventional components, sometimes at multiple levels, and are usually embedded within the context of their application (Nielsen and Abildgaard 2013; Nielsen and Miraglia 2017; Montano et al. 2014). However, evaluations of the effect of these interventions have been inconclusive (Gray et al. 2019; Montano et al. 2014; Ruotsalainen et al. 2015; Semmer 2006). Thus, it is important to understand both how to design and implement organizational-level workplace interventions to attain maximum impact on the organization (Aust et al. 2023; von Thiele Schwarz et al. 2021). Qualitative process data can be valuable in developing this understanding and to achieve a sustainable and desired intervention effect (Egan et al. 2007; Kristensen 2005; Nielsen and Randall 2013; Nielsen et al. 2010). There is also a methodological challenge in how to combine qualitative and quantitative data in mixed-method designs with the purpose to evaluate how and why an intervention does or does not work (Greasley and Edwards 2015; Härenstam et al. 2019; Nielsen and Abildgaard 2013).

Previous process evaluations of organizational-level interventions have shown that several factors are important to achieve the desired outcomes of the intervention (for instance, see Herrera-Sánchez et al. 2017; von Thiele Schwarz et al. 2021), such as having support from the management (Härenstam et al. 2019; LaMontagne et al. 2012), ensuring high participation from the target group (Nielsen and Christensen 2021), and fitting the intervention to the organizational context (Ipsen et al. 2015). The later has been identified as an important factor for the success of organizational-level interventions and the absence of co-occurring changes in combination with managerial support or a combination of role clarity, employee participation, and team learning has been associated with increased intervention fit, while limited leader support, low degree of role clarity, or concurrent organizational changes have been associated with decreased intervention fit (Roczniewska et al 2023). While multiple factors that may facilitate or hamper successful interventions have been identified, it is unclear which of them that are sufficient (i.e., enough by themselves but not the only path to the outcome), which are necessary (i.e., always must be present), and which are both necessary and sufficient to produce the desired intervention effect. Yet, such knowledge seems crucial to those who design and implement interventions, especially when resources are scarce. Thus, we need more research into the difference-making factors, i.e., those that consistently distinguish cases with an outcome of interest from those without.

The aim of this study was to analyze data from a large-scale organizational-level intervention to identify key difference-making factors that consistently and uniquely distinguished intervention groups with a positive intervention effect on sickness absence from those groups that did not have a positive intervention effect.

## Methods

### Setting and intervention design

The present study was carried out between 2017 and 2018 in one of the 21 Swedish healthcare regions with about 56,000 public-sector employees. Between 2017 and 2018, an organizational-level intervention was launched to improve working conditions and decreasing sickness absence among their employees. The intervention was designed to address organizational-level causes of the problems, rather than employee behaviors.

The intervention has been described in detail elsewhere (Akerstrom et al. 2021; Wikström et al. 2021). Briefly, an in-depth analysis of the sickness absence and employee turnover was performed within the healthcare region. Eight operational areas (one of the organization’s hierarchical levels, consisting of departments, operational areas, and workplaces) were identified as having high sickness absence (> 10%, chosen pragmatically, where the regions’ average total sickness absence varied between 5.5 and 6.8% from 2013 to 2019) in combination with a high employee turnover (qualitative assessment to verify that the identified operational areas needed support). The management of these operational areas was contacted and invited to participate in this organizational-level workplace intervention. All invited operational areas gave their informed consent to participate and an appropriate intervention group was selected within each operational area by the management of the operational area. An external process facilitator was assigned to each intervention group to improve implementation and adherence to the intention underlying the intervention. As a part of the intervention, a strategic group was formed, including managers on more than one hierarchical level within the organization and representatives from human resources (HR) and other stakeholders. With support from the facilitator, the role of the strategic group was to identify group-specific causes of the challenges within the workplace, suggest measures to address these causes, and implement the suggested measures to ensure a good fit between the interventional measures and the local context. Interventional measures were intended to affect the employees’ work environment, preferably by targeting the “causes of the causes”, i.e., how work was organized and/or executed, rather than targeting individual employees (Akerstrom et al. 2021). The intervention process and effects were evaluated as an externally funded project, separate from the intervention, where the effect evaluation showed an overall positive intervention effect on sickness absence, but the intervention effect varied greatly between the eight intervention groups (Akerstrom et al. 2021). The process evaluation identified several supporting conditions during the implementation process, such as central foci, sense of urgency, knowledge of work environment management, and change process management. The presence of these supporting conditions could partially explain the variation in the intervention effect (Wikström et al. 2021).

### Study population

All participating intervention groups operated within healthcare. Six of the eight groups primarily cared for patients within pediatric care (*n* = 2), psychiatric care (*n* = 2), orthopedic surgery (*n* = 1), and radiology (*n* = 1). The remaining two intervention groups provided support within hospital service and maintenance, and orthopedic aid and sterilization units. Together, the eight selected groups comprised of about 1640 employees with a mean of 205 employees per intervention group (range 41–458).

### Qualitative process evaluation

The process evaluation was performed using process and contextual information provided from the process facilitators and/or administrative personnel systems within the region (Akerstrom et al. 2021; Wikström et al. 2021). For each intervention group, a non-standardized log was kept by the process facilitator during the intervention. These logs comprised of about 30–40 pages each and contained documentation of the progress and important events or incidents (meetings, decisions, changes in key personnel, etc.). The logs were compiled in a standardized format based on the fixed categories: background, challenge, goal, course of events, initiation, context and sense of urgency, strategic group, measures (discussed, planned, and implemented), critical incidents, key roles, and the facilitator’s perception of the process. This standardized process documentation was then analyzed using a thematic approach (Braun and Clarke 2006; Miles et al. 2014) to identify facilitating and hindering conditions during the implementation process. In addition, each process facilitator provided group-specific contextual information and qualitative reflections on the process and the intervention groups´ adherence to the intention of the intervention in structured group interviews.

### Quantitative effect evaluation

In the effectiveness evaluation (Akerstrom et al. 2021), monthly data from the region’s administrative personnel system between January 2015 and October 2019 (i.e., covering the pre-, intervention [about 2 years] and post-intervention phases for the individual intervention processes) were obtained for each of the eight intervention groups. The overall intervention effect on sickness absence was estimated for three sub-groups of the participating workplaces, grouped according to their individual performance in the implementation process (Wikström et al. 2021) using a random-intercept or random-coefficient model (PROC MIXED in SAS version 9.4; SAS Institute, Cary, NC, USA) with group and time (nested within group) as random effects. In addition, a first-order autoregressive correlation structure (AR [1]) was used to account for correlations between repeated measurements of the same group. Fixed effects for year (continuous) and month (categorical 1–12) were added to the model to control for time trends and seasonality, and a dummy variable for the intervention (0 up to the beginning of the intervention process and then 1) was added to analyze the effect of the intervention.

### Contextual and processual factors and intervention outcomes

In total, 34 different contextual and processual factors were retrieved from the process and effect evaluations. Four factors had low variation, i.e., single intervention groups represented one or more categories, and were consequently excluded, resulting in 28 remaining factors (see Table 1). Two outcomes retrieved from the process and effect evaluations were used: having a successful implementation of tailored interventional measures on an organizational level (yes or no) and the intervention effect on sickness absence from the quantitative effect evaluation, categorized as no effect (overall effect − 0.24, 95% confidence interval (CI) − 1.9–1.5, *p* = 0.8), medium effect (overall effect − 1.3, 95% CI − 2.6–0.002, *p* = 0.05), and high effect (overall effect − 4.2, 95% CI − 5.9 to − 2.4, *p* < 0.001) (Table 1). The first outcome was assessed in the qualitative process evaluation (Wikström et al. 2021) using focus group interviews with the process facilitators that had been involved in all phases of the respective intervention groups. A successful implementation of tailored interventional measures on an organizational level was defined as having identified intervention measures on an organizational level tailored to the specific needs of the intervention group (in the design phase of the intervention) and implementing these measures in the implementation phase of the intervention. The second outcome was derived from the quantitative effect evaluation (Akerstrom et al 2021; Wikström et al. 2021) according to above.

**Table 1:** Contextual and processual factors, and outcome measures from the process and effect evaluation, and their calibration

Factors	Categories and calibration	Justification^a^
*Contextual*		
Type of workplace	1 = orthopedic surgery & radiology, 2 = psychiatric care, 3 = pediatric care, 4 = service units	1
Size of the intervention group (n)	1 = < 100, 2 = 100–199, 3 = 200–299, 4 = ≥ 300	2
Organizational complexity of the intervention group	0 = no (1–2 units), 1 = yes (< 3 units)	2
Identified unit affected but not included in the intervention	0 = no, 1 = yes	3
Size of operational area (n)	1 = < 299, 2 = 300–2000, 3 = > 2000	2
Size of department (n)	1 = < 500 employees, 2 = 500–5000 employees, 3 = > 50,000 employees	2
Gender distribution in the intervention group (%)	1 = < 15, 2 = ≥ 15	2
Sickness absence 12 months before the intervention (%)	1 = < 10, 2 = 10–15, 3 = > 15	2
Employee turnover 12 months before the intervention (%)	1 = < 1.0, 2 = ≥ 1.0	2
Shared understanding sustainable work processes	0 = no, 1 = yes	3
The intervention group had good knowledge of social and organizational work environment management	0 = no, 1 = yes	3
The intervention group had good process knowledge and good experiences of driving change processes and of implementing new working methods	0 = no, 1 = yes	3
Presence of good cooperation and trust between occupational health services, HR, and managers prior to the intervention	0 = no, 1 = yes	3
*Processual*		
Time length between initiation and first strategic meeting	1 = short, 2 = long	3
Time length between initiation to first implemented interventional measure	1 = short, 2 = long	3
The organizational unit themselves, before they were contacted by the facilitating role and process support, had made problem analyzes	0 = no, 1 = yes	3
The organizational unit perceived that the work environment needed to change and wanted support	0 = no, 1 = yes	3
Participation from top management in formulating the problems	0 = no, 1 = yes	3
Participation from employees in formulating the problems	0 = no, 1 = yes	3
Good anchoring and the strategic management level participates	0 = no, 1 = yes	3
High sense of urgency	0 = no, 1 = yes	3
Key actor	1 = manager, 2 = no manager	3
Action plan was setup	0 = no, 1 = yes	3
Action plans and interventional measures targeted organizational conditions	0 = no, 1 = yes	3
On site commitment in the implementation phase including inclusion of employees	0 = no, 1 = yes	3
Need for more than one restart of the intervention	0 = no, 1 = yes	3
Manager turnover during the intervention	0 = no, 1 = yes—one, 2 = yes—two	4
Process facilitator turnover during the intervention	0 = no, 1 = yes—one, 2 = yes—two	3
Number of meetings in strategic group	1 = low (< 5), 2 = high (≥ 5)	2
Intervention cost per employee	1 = < 3000, 2 = 3000–5000, 3 = > 5000	2
Changes in strategic group	0 = no, 1 = yes	3
Adherence to the intention of the intervention	0 = low, 1 = high	3
*Outcomes*		
Size of intervention effect sickness absence	0 = none^b^, 1 = medium^c^, 2 = high^d^	4
A successful implementation of tailored interventional measures on an organizational level	0 = no, 1 = yes	3

^a^1 = Theoretical assumptions on similar contexts; 2 = Natural gaps in data; 3 = Binary categories from the qualitative process evaluation; 4 = Categories from the quantitative process evaluation

^b^overall effect − 0.24, 95% confidence interval (CI) − 1.9–1.5, *p* = 0.8

^c^overall effect − 1.3, 95% CI − 26–0.002, *p* = 0.05

^d^overall effect − 4.2, 95% CI − 5.9 to − 2.4, *p* < 0.001

### Analytical approach

We used Coincidence Analysis (CNA) to identify difference-making factors that uniquely distinguished cases with and without the outcome (Whitaker et al. 2020). CNA is a relatively new case-based, mathematical approach to data analysis that draws on Boolean algebra, set theory, and formal logic. The algorithm at the foundation of the R package “cna” is custom designed to address both causal complexity and equifinality. Causal complexity is when specific combinations of factors together explain an outcome, whereas equifinality is when multiple paths lead to the same outcome (Baumgartner et al. 2009; Whitaker et al. 2020). Importantly for this study, CNA does not require large sample sizes to achieve valid results and may be applied to small sample studies as an analytical method to study implementation (Adams et al. 2022; Damschroder et al. 2022; Petrik et al. 2020; Rattray et al. 2023; Sperber et al. 2022; Whitaker et al. 2020; Womack et al. 2022). Thus, a mixed-method approach using CNA offers a novel way to identify specific organizational factors that are necessary and sufficient for implementation success.

In the analysis, the steps outlined in Whitaker et al. (2020) along with using the “msc” routine for factor selection were followed (Roczniewska et al 2023). In the first step, the outcomes and predictors, derived from the qualitative process evaluation and the quantitative effect evaluation, were calibrated, i.e., assigned categorical values to each case for each variable. These decisions were made based on either binary categories (for instance yes/no) or up to four different categories based on theoretical assumptions or natural gaps in data (for instance low/medium/high). Table 1 demonstrates decisions and thresholds for calibration for all variables, as well as their justification.

Because the number of cases was relatively small compared to the number of factors, the second step in our preparatory analyses involved data reduction using a configurational approach described in detail in prior literature (Damschroder et al. 2022; Miech et al. 2022; Rich et al. 2022; Roczniewska et al 2023; Yakovchenko et al. 2020). Specifically, we applied the minimally sufficient conditions (msc) routine within the “cna" package in R across the entire dataset to identify specific configurations of conditions strongly linked to the outcomes of interest (i.e., having a successful implementation process and the intervention effect on sickness absence). In this process, we exhaustively considered all possible combinations of one-, two-, and three-condition configurations in the data, retained all configurations that met the prespecified consistency threshold, and then generated a “condition table” to organize the Boolean output. In a condition table, rows list all configurations of conditions meeting the specified consistency level, with separate columns for outcome, conditions, consistency, and coverage. Consistency is a measure of model reliability calculated as the number of intervention groups consistent with the model where the outcome is present divided by the total number of intervention groups where that model is present. Coverage is a measure of explanatory breadth; it represents the number of intervention groups covered by the model where the outcome is present, divided by the total number of intervention groups with the outcome present. When initiating the msc routine, we first specified a consistency threshold of 100% and if no configurations met this threshold, we lowered the specified consistency level by 5 percentage points (e.g., from 100 to 95%, etc.) and repeated the process to generate a new condition table. We continued to lower the consistency threshold until all the following criteria were met:

- “Best of class” coverage scores (i.e., top coverage score among configurations with the same number of conditions)
-  ≥ 1 mutable condition in each candidate configuration (to ensure relevance to research question)
- Candidate configurations consistent with logic, theory, and prior knowledge
- The same set of factors distinguish different levels of the outcome when taking on different factor values (i.e., the outcome changes when these difference-making factors take on different values).

Using this approach, we inductively analyzed the entire dataset and used the condition table output to identify a subset of candidate factors for model development during the next step of configurational analysis.

In the second step, we proceeded to the modeling phase of CNA, where the goal was to produce a model which explained at least 80% of the intervention groups with the outcome (coverage), yield the outcome at least 80% of the time the solution appeared anywhere in the dataset (consistency), and there was only one solution (Baumgartner and Ambuhl 2018).

Data reduction and subsequent model development were conducted separately for the two outcomes. To ensure that models were not influenced by one or two intervention groups, we also performed sensitivity analyses for each outcome where we evaluated any differences in the models after making each of the following changes: (1) removing each of the eight intervention groups one at a time (8 different possibilities for each outcome) and (2) removing two intervention groups at once from two different outcome levels (12 different possibilities for the “intervention success” outcome and 20 different possibilities for the “effect on sickness absence” outcome).

## Results

CNA analyses were performed to identify difference-making factors that distinguish intervention groups with and without a positive intervention effect. The analysis was performed separately for two outcomes, a successful implementation of tailored interventional measures on an organizational level and the intervention effect on sickness absence. The models for the two outcomes are visualized in Tables 2 and 3, respectively. Both models had coverage and consistency scores of 100%.

**Table 2:** Final model from coincidence analysis on the difference-making factors for a successful implementation of tailored interventional measures on an organizational level

Intervention group	Outcome variable	Process factors	
	A successful implementation of tailored interventional measures on an organizational level	High sense of urgency	Good anchoring and participation from strategic management^a^
1	Yes	Yes	Yes
2		Yes	Yes
3		No	Yes
5		Yes	Yes
6		Yes	No
8		Yes	No
4	No	No	No
7		No	No

Overall model consistency = 100%; overall model coverage = 100%

^a^The management clearly supporting and positioning this initiative within the larger mission, vision and values of the organization

**Table 3:** Final model from coincidence analysis on the difference-making factors for the intervention effect on sickness absence

Intervention group	Outcome variable	Process factors	
	Intervention effect on sickness absence	A successful implementation of tailored interventional measures on an organizational level	High employee participation during the implementation phase
1	High	Yes	Yes
2		Yes	Yes
3	Medium	Yes	No
5		Yes	No
6		Yes	No
8		Yes	No
4	None	No	No
7		No	No

Overall model consistency = 100%; overall model coverage = 100%

For the first outcome, we modeled the presence and absence of having a successful implementation of tailored interventional measures on an organizational level. The result showed that there were two paths to achieving a successful implementation; if either a high sense of urgency or good anchoring and participation from the strategic management (i.e., the management clearly supporting and positioning this initiative within the larger mission, vision and values of the organization) was present, then a successful implementation was achieved. When neither of these conditions were present, then a successful implementation of tailored interventional measures on an organizational level was not observed (Table 2).

In 3 of the 20 sensitivity analyses (15%) conducted for this outcome, if Intervention Group 3 was removed (Table 2), the model simplified to a single factor: the presence or absence of high sense of urgency.

For the second outcome, we aimed to model three levels of the intervention effect on sickness absence (high, medium, and none) derived from the quantitative analyses. The results showed that the presence of high employee participation during the implementation phase by itself was necessary and sufficient for a high intervention effect (Table 3). For organizations without high employee participation, then the presence of a successful implementation consistently led to the intervention having a medium intervention effect. Organizations that had neither high employee participation nor successful implementation were sites with no intervention effect on sickness absence.

This model remained unchanged after conducting all 28 sensitivity analyses for this outcome.

## Discussion

In this study, a configurational approach using CNA was applied to identify difference-making factors that uniquely distinguished workplaces with and without a successful implementation and a high intervention effect in an organizational-level intervention in the public sector of Sweden. The results indicate a potential two-stage mechanism where either a high sense of urgency or a good anchoring and participation from the strategic management (i.e., the management clearly supporting and positioning this initiative within the larger mission, vision and values of the organization) needs to be present to achieve successful implementation of tailored interventional measures on an organizational level, and a positive intervention effect on the sickness absence among the employees. However, high participation from the strategic management was not sufficient for a maximum impact, as high participation among employees during the implementation phase was also necessary to gain a high intervention effect on the sickness absence.

The importance of participation when performing organizational-level workplace intervention has been shown repeatedly in the past (Abildgaard et al. 2020; Aust et al. 2023; Fox et al. 2022; von Thiele Schwartz et al. 2021) and participatory approaches have consequently been recommended (Nielsen and Miraglia 2017). A high participation will improve the design of the intervention by increasing the fit to the context (Aust et al. 2023; McFillen et al. 2013; Nielsen et al. 2015; Storkholm et al. 2019), increase the participants´ commitment to the intervention (Rosskam 2018), and better align the intervention into the existing work practices and procedures (Tsutsumi et al. 2009). However, what is meant by participation varies greatly (Abildgaard et al. 2020) and our findings shed some light on what kind of participation needs to be present in different phases of the intervention to maximize the effect of an organizational-level intervention.

When it comes to the participation of the management, the line management has traditionally been described as key actors (Hasson et al. 2014), but our results show the importance of the involvement of the strategic management, as well. Our results also stress that strategic management participation cannot be created solely by forming a group including representatives from the top management to secure a successful implementation. They also need to exhibit an active engagement and a sense of urgency; in fact, when Intervention Group 3 was removed as part of the sensitivity analyses, the model simplified to this single factor, underscoring its importance. In our study, four out of eight workplaces were found to have a good anchoring and participation from the top management despite that all workplaces had a strategic group consisting of representatives from the strategic management and only five out of eight workplaces experienced a high sense of urgency. Thus, the organization needs to build a capacity for enabling a successful intervention in the pre-intervention phase ensuring both an active engagement and participation within managers on different levels and a mutual understanding of the situation and the objectives of the intervention (von Thiele Schwarz et al. 2021).

While key difference-making factors for a successful implementation were largely found on the managerial level, the participation among the employees was found to be important for maximizing the intervention effect. In a recent systematic review of organizational-level interventions, employees influence and participation in workplace intervention have been found to play a central role (Aust et al. 2023). Like the participation of the management, this study shows that a high employee participation will improve the design of the intervention, add to a mutual understanding, ensure a good fit to the context, and increase the commitment to the intervention. Furthermore, employee participation will also increase employees´ influence on the way work is organized and increase job control which have been seen to both prevent burnout and promote job satisfaction (O’Connor et al. 2018; Zangaro and Soeken 2007).

It is also important to consider the interplay between managers’ and employee participation. A recent systematic review on the effectiveness on organizational- and group-level interventions on employee well-being concluded that interventions where the management create opportunities for workers to participate through feedback and process modifications throughout the implementation process was particularly effective. Contrary, if the management had a stronger focus on increased productivity or higher quality standards in the intervention, rather than provide opportunities for workers participation, failure to improve well-being was more often evident (Fox et al. 2022).

There is a challenge with generalizing and transferring organizational-level workplace interventions between organizations, since they typically are tailored to a specific context. To overcome these challenges, adaptions to the intervention need to be done in the implementation process to align the intervention with the existing organizational objectives and create a good fit to the context. The literature offers general recommendations and practical guidelines for conducting organizational-level interventions (Herrera-Sánchez et al. 2017; von Thiele Schwartz et al. 2021), but there is a lack of knowledge whether all these recommendations need to be followed to achieve the desired outcomes of the intervention. It has been suggested that as many supporting factors as possible need to be in place for a successful implementation but the degree to which it is feasible to do so differ between occasions and contexts (von Thiele Schwartz et al. 2021). By reanalyzing data from these well-documented and investigated cases, it was possible to move beyond supporting factors and investigate what specific preconditions for the investigation that were necessary for a positive outcome and to what extent failures could be attributed to the absence of these necessary preconditions. In our case, nine supportive factors affecting the intervention effect had been earlier identified in a conventional process evaluation (Wikström et al. 2021) and this current re-analysis, three difference-making factors were identified among these: two factors explained a successful implementation and the presence or absence of an intervention effect, and another factor explained the level of success. However, this does not necessarily mean that these three difference-making factors are the only factors that matter. For example, it may be the case that if there is a high participation on multiple levels within an organization, one could expect a high overall capacity for change with also other supporting factors in place. This might also be a reason for that other, well-known supportive factors in this study did not remain in the final models, since they were not necessary and sufficient on their own. The role and relative importance of these three factors in other context and other organizational-level interventions warrants further investigation.

### Strengths and limitations

A strength of this study is its mixed-methods approach using high-quality data including the extensive process documentation created by the external process facilitators during the implementation process, group-specific contextual information, and qualitative reflections on the process and the intervention groups’ adherence to the intention of the intervention collected in structured focus group interviews and register data on sickness absence provided by the employer.

A case-based method has been used in the analyses on these eight interventions groups within a single organization within the public sector; these findings may not automatically generalize to other organizations. In addition, the limited number of intervention groups in this study may also affect the possibility to generalize the results. However, as this process evaluation investigates the use of process facilitators within the public sector rather than an organization-specific intervention, these findings may prove relevant in other contexts.

Finally, the intervention effect on sickness absence was categorized using results from a previous effect evaluation and categorized as none, medium, and high effect. All groups receiving the intervention were selected in the first place, because they experienced high sickness absence in combination with high employee turnover, which may have affected the magnitude of change. Accordingly, since the magnitude of an intervention effect needs to be assessed in relation to its context, the difference-making factors may have explained relative level of success rather than a high intervention effect (Tanner-Smith et al. 2018).

## Conclusions

This study identified two types of participation as difference-making factors in the implementation process from a much larger pool of candidate factors consisting of contextual and processual factors from an organizational-level intervention within the Swedish public sector. A possible mechanism was seen where either a high sense of urgency or a good anchoring with participation from the strategic management (i.e., the management clearly supporting and positioning this initiative within the larger mission, vision and values of the organization) was found to be sufficient for successful implementation. Furthermore, only the presence of high participation from employees during the implementation phase alone explained a substantial drop in sickness absence. Thus, when implementing organizational-level interventions, high participation from the strategic management and the employees are both needed at different phases to maximize the intervention effect on sickness absence.

## Data Availability

The datasets generated during and/or analyzed during the current study are available from the corresponding author on reasonable request.
